# Healthcare Professionals’ perspectives on AI-driven decision support in young adult mental health: an analysis through the lens of a shared decision-making framework

**DOI:** 10.3389/fdgth.2025.1588759

**Published:** 2025-09-25

**Authors:** Hassan Auf, Jens Nygren, Lina E. Lundgren, Lena Petersson, Petra Svedberg

**Affiliations:** ^1^ School of Health and Welfare, Halmstad University, Halmstad, Sweden; ^2^ School of Business, Innovation and Sustainability, Halmstad University, Halmstad, Sweden

**Keywords:** artificial intelligence, mental health, shared decision making, decision support system, healthcare professionals, young adults, healthcare

## Abstract

**Background:**

Mental healthcare faces growing challenges due to rising mental health issues, particularly among young adults. AI-based systems show promise in supporting prevention, diagnosis, and treatment through personalized care but raise concerns about trust, inclusivity, and workflow integration. Limited research exists on aligning AI functionalities with healthcare professionals’ needs or incorporating shared decision-making (SDM) into AI-supported mental health services, emphasizing the need for further exploration.

**Objective:**

This study aims to explore how AI-based decision support systems can be used in mental healthcare from the perspective of healthcare professionals and in the light of a SDM framework.

**Methods:**

A qualitative approach using deductive content analysis was employed. Sixteen healthcare professionals working with young adults participated in semi-structured interviews. The analysis was guided by elements of SDM to identify key needs and concerns related to AI.

**Results:**

Healthcare professionals acknowledged both the potential benefits and challenges of integrating AI-based decision support systems into SDM for mental healthcare. Fifteen of 23 SDM elements were identified as relevant. AI was valued for its potential in early detection, holistic assessments, and personalized treatment recommendations. However, concerns were raised about inaccuracies in interpreting non-verbal cues, risks of overdiagnosis, reduced clinician autonomy, and weakened trust and therapeutic relationships.

**Conclusions:**

AI holds promise for enhancing triage, patient participation, and information exchange in mental healthcare. However, concerns about trust, safety, and overreliance on technology must be addressed. Future efforts should prioritize human-centric SDM, ensuring AI implementation mitigates risks related to equity, data privacy, and the preservation of therapeutic relationships.

## 1 Introduction

Mental healthcare faces significant challenges due to the increasing prevalence of mental health issues over recent decades in Sweden, as in many other European countries (1–3). These challenges exert considerable strain on healthcare systems, escalating both health and economic demands as they strive to support affected individuals while maintaining essential services. Mental health disorders account for approximately 31% of the total Disability-Adjusted Life Years (DALYs) among individuals aged 10–24 years (4). This burden continues to rise with no indications of declining (5–7).

Around 75% of all mental disorders have their onset during adolescence and early adulthood, typically before the age of 25 (8). Despite this, young adult with mild to moderate symptoms often face longer waiting times and receive lower prioritization than those with more severe conditions, which can potentially lead to progression of their problems (9, 10). Delays in early intervention and treatment are particularly concerning given the recurring episodes of mental health problems in this age group (11). Such delays substantially increase the likelihood of adverse long-term outcomes and elevate the risk of developing complex, chronic mental health disorders in adulthood (11–13).

Healthcare systems in Sweden and European countries face significant challenges in addressing young adult's mental health needs. Barriers include long waiting time inadequate service provision, and limited accessibility to appropriate care (10, 14, 15). Research in Sweden indicates that young adults often struggle to access help due to limited awareness of mental health issues and insufficient availability of services, As a result, many attempt to cope independently (14, 16, 17) Similarly, in the UK, wait times for mental health services can extend up to 18 weeks (18). Given the rising demand and the insufficient readiness of many healthcare systems, research underscores the necessity for governments to prioritize investment in mental healthcare services (19). Enhancing support for young people is essential to reduce the long-term burden on healthcare systems and to improve mental health outcomes at both individual and societal levels (20).

There is growing interest in using digital systems powered by artificial intelligence (AI) in response to these demands in order to support healthcare professionals and patients throughout the mental healthcare continuum, including prevention, diagnosis, and treatment (21, 22). AI has been recognized for its potential to enhance personalized care, promote sustainable mental healthcare by aligning interventions with the unique needs and preferences of individual patients (23). Various AI systems are being developed to support decision-making in mental health; however, research examining their impact on clinician-patient interactions, trust, and acceptance remains limited. Existing studies highlight these factors as potential barriers that may pose challenges on the implementation and sustainable use of AI systems (24–26). Concerns have been raised regarding clinician-patient interactions when AI is used in healthcare (24, 27). Key risks include reduced patient inclusivity, decision paralysis among clinicians uncertain about AI recommendations, and communication challenges—both in conveying AI outputs to patients and integrating patient input. Ambiguity around the roles of AI and clinicians may also undermine patient trust and the therapeutic relationship. Addressing these issues proactively is crucial to ensure safe and effective AI integration in mental health care.

Healthcare decision-making has progressively evolved to actively involve patients in their care processes. Numerous studies highlight the benefits of transitioning from disease-centered care to person-centered care (28, 29). Shared decision-making (SDM) embodies this concept by engaging healthcare professionals and patients collaboratively in decision-making processes. SDM is defined as an approach in which clinicians and patients share the best available evidence to make decisions, with patients receiving support to evaluate options and achieve informed preferences (30). Implementation of SDM in the mental healthcare involve various strategies, techniques, and methods, including patient decision support aids, patient education, and the development of healthcare professional's communication techniques and personal skills to include the patient in the decision-making process (31). Research also suggests that decision aids, which actively invite users to express their goals and consider alternatives, can help legitimize and incorporate their knowledge and preferences into the decision-making process (32). However, for these methods to be effective, SDM needs to be systematically planned and integrated into healthcare environments and services. This includes assessing the specific needs for SDM to ensure alignment with both end-user preferences and organizational goals (33, 34). By providing relevant, on-demand information and facilitating interactions, modern technologies, including AI systems, have the potential to strengthen such collaborative decision-making (35). However, there is limited literature describing the use and implementation of AI-based decision support systems within healthcare, particularly in mental health and in relation to SDM (36, 37).

While SDM provides a framework for structuring collaborative decision-making in healthcare, the integration of AI introduces both new opportunities and challenges. Human–AI collaboration illustrates how clinical expertise can be complemented by machine intelligence to strengthen decision quality, improve outcomes, and increase productivity (38, 39). Yet risks remain, particularly algorithmic bias, which has led in practice to large-scale inequities such as patients being excluded from access to care (40). Approaches such as human-centered AI and human–AI teaming aim to mitigate these risks by embedding human needs, preferences, and trustworthiness in system design, while promoting the complementary strengths of humans and AI (41, 80). In mental health, such approaches have shown promises in supporting user motivation, aiding interpretation of AI outputs, and preserving agency in decision-making (42), thereby offering a pathway for integrating AI into SDM practices in ways that enhance rather than undermine equity and collaboration.

While AI-based decision support systems hold considerable promise for enhancing decision-making in mental healthcare, their successful implementation may be challenged by the disconnection between healthcare professionals' needs and the practical utility of AI systems, resulting in barriers to the clinical workflows and potential engagement issues in practice (25, 43). Current research on introducing AI for decision support in mental health services remains in its infancy (44, 45), with most studies focusing on pre-implementation phases rather than real-world clinical applications (45). Notably, the integration of SDM in AI-based decision support systems remains unexamined despite its critical role in person-centered care (45).

Given these gaps, it is crucial to investigate the perspectives of healthcare professionals on AI-based decision support in mental health, particularly regarding their requirements related to SDM. This study aims to explore how AI-based decision support systems can be used in mental healthcare, especially for young adults, from the perspective of healthcare professionals and in the light of an SDM framework.

## 2 Methods

### 2.1 Study design

This study employed a qualitative design, utilizing a deductive approach to qualitative content analysis (46, 47). A deductive approach was chosen to incorporate the key elements of SDM as predefined categories for analysis (48). The study adheres to the 32-item Consolidated Criteria for Reporting Qualitative Research (COREQ) to ensure rigor and trustworthiness in the qualitative analysis (49).

### 2.2 Theoretical framework

The SDM framework proposed by Makoul and Clayman (48) was adopted as the theoretical foundation for this study. This framework categorizes SDM elements into three types: essential, ideal, and general (see Supplementary Table S1). For each element, we have further clarified and expanded the definitions within the proposed SDM framework, drawing on additional references to enhance comprehensiveness.

• Essential elements represent the practical or clinically oriented steps of SDM necessary for interactions between healthcare professionals and patients. These elements include defining the problem, presenting treatment options, discussing pros and cons, eliciting patient values and preferences, evaluating patient self-efficacy, providing clinician recommendations, clarifying understanding, explicitly making or deferring decisions, and arranging follow-up.
• Ideal elements enhance SDM practice but are not mandatory, such as defining roles, presenting evidence, ensuring unbiased information, and reaching mutual agreement.
• General elements emphasize value-based approaches and focus on the overall SDM process and quality rather than specific practical behaviors. These include deliberation, individualized approaches, information exchange, mutual respect, partnership, patient participation, and patient education, with broader applications throughout the care journey.

### 2.3 Setting and participants

Young adults with common mental health problems can turn to a variety of healthcare settings in the Swedish healthcare system such as primary care units, youth guidance centers, and student healthcare services (50). Thus, care for patients with common mental health problems in Sweden is traditionally provided by healthcare professionals outside of specialized care services such as psychiatry and these healthcare settings are regarded as first line of care in Sweden. All participants in the current study work outside of the specialized care services and were recruited based on their professional experience working with young adults aged 18–30 presenting with common mental health problems. No requirements for inclusion were set regarding formal training or prior knowledge of SDM practices, and such information was not specifically at the scope of the study's data collection. A snowball sampling procedure was employed in a variety of healthcare settings (49). A purposive selection of individuals meeting the study's inclusion criteria was initially made within each clinical setting. These individuals subsequently recommended additional participants from their professional networks.

A total of 15 healthcare professionals participated in semi-structured interviews. The sample consisted of psychologists (*N* = 6), nurses (*N* = 5), welfare officers (*N* = 3), and a social worker (*N* = 1) who meet young adults in their daily clinical work. The gender distribution comprised 10 females and 5 males. Participants were drawn from various clinical settings in first line of psychiatric care, including primary care (*n* = 9), youth guidance centers (*n* = 4), and student affairs support (*n* = 2).

### 2.4 Data collection

The data collection was carried out in semi-structured individual interviews conducted either in person or digitally by researchers with previous interviewing experience (JMS, KHW and MA) between October 2020 and February 2023. The researchers had no previous relationships with the participants. The interview durations ranged from 34 to 57 min, amounting to a total of 11 h and 56 min. All the interviews were audio-recorded and transcribed verbatim. The interviews were based on an interview guide that focused on the use of AI technology as decision support in implementing interventions aimed at delivering integrated and accessible care for young adults with mental health issues. The guide included questions such as: (1) the informant's role and prior experience in working with mental illness and in applying AI-based technologies in clinical practice; (2) the informant's perspectives on mental illness, help-seeking behavior, and the provision of preventive interventions; (3) perceived opportunities and challenges that must be addressed to establish coherent, accessible, and person-centered care for young individuals with mental health needs; and (4) attitudes toward the potential use of AI-based technology to support clinical decision-making; (5) obstacles, opportunities and facilitating factors that needs to be taken into account for the introduction of new AI based methods/interventions to fit into existing processes and systems. To encourage participants to share more in-depth and reflective responses, follow-up prompts were used, such as “Could you elaborate on that?”, “Can you explain what you meant?”, and “Can you give an example?”. These open-ended prompts aimed to create space for deeper exploration and richer narratives.

### 2.5 Data analysis

A deductive content analysis, as described by Graneheim et al. (46, 81), was conducted to explore the healthcare professionals' perspectives on how AI systems could support SDM in mental healthcare. The analysis employed the SDM framework by Makoul and Clayman (48) to ensure a thorough understanding of how AI systems can be used in practice to support healthcare professionals in relation to SDM. A codebook was developed to guide the extraction and analysis of meaning units, incorporating definitions, operational descriptions of SDM elements, and extraction probes. It was based on the 23 SDM elements described by Makoul and Clayman (48) which each element assigned a definition extracted from relevant SDM literature (see Supplementary Table S1). To maintain consistency in interpretation, each element was further provided with description for its operational use and specific identification probes. The approach and structure of the codebook were collaboratively discussed and refined by the authors, to improve reliability and coherence.

The first author (HA) read all the transcripts multiple times to ensure familiarity with the content. The meaning units related to participants' needs and concerns regarding SDM elements were then identified through a deductive approach and then abstracted, organized, and analyzed to describe the healthcare professionals' requirements. Throughout the analytical process, all authors (HA, PS, JN, LL) were engaged in iterative discussions and critical reflections, allowing for refinement of categories, resolution of interpretive differences, and validation of analytical decisions. This collaborative and iterative process of abstracting meaning units, organizing them into elements, and conducting analysis helped ensure that no meaningful data were overlooked and contributed to the credibility and depth of the analysis. The approach aligns with the guidance of Graneheim et al. (46), who emphasize the value of reflexivity and researcher dialogue in ensuring rigor in qualitative content analysis. To further strengthen trustworthiness, the healthcare professionals' quotations used in this paper were translated from Swedish to English by a native English-speaking professional proofreader and were edited only slightly to improve readability.

### 2.6 Ethical considerations

The Swedish Ethical Review Authority (2020-06246) approved the project. Participants signed an informed consent before entering the study. Participation was voluntary, and participants could withdraw without stating a reason. The study adhered to the guidelines in the Declaration of Helsinki (51).

## 3 Results

The healthcare professionals provided their perspectives on AI-based decision support systems in mental health, specifically addressing their needs and concerns related to the SDM elements. As a result, 15 of the 23 SDM elements were identified within the three categories of SDM (essential, ideal, and general). Supplementary Table S2 summarizes the coding framework, indicating for each element whether and to what extent it was present in the data. The participants' perspectives on the SDM elements in the three categories were as follows, exemplified by citations translated from Swedish to English.

### 3.1 Essential elements

This category highlights needs and concerns primarily associated with four of the nine essential elements of SDM: (1) defining/explaining the problem, (2) patient values/preferences, (3) healthcare professional knowledge/recommendations, and (4) arranging follow-up.

#### 3.1.1 Defining/explaining the problem

Healthcare professionals emphasized the potential for AI to aid in the early detection of common mental health challenges, monitor the progression of mental health conditions, and identify preventive measures and suicide risk factors. Participants expressed a desire for AI systems to support a more holistic understanding of mental health problems by enabling open-ended and needs-focused assessments rather than the rigid question-and-answer forms currently used. They also advocated for AI to incorporate lifestyle factors when evaluating mental health problems and severity, requesting to improve triage accuracy while empowering patients to articulate their problems in their own words.

“then there will be a contact form, which isn’t a depression form but more “can you describe your symptoms in more detail? How would you rate your anxiety? Do you have any suicidal thoughts? Any previous suicide attempts? What are your concerns? What are you worried about? What help do you hope to get?” and such like. And if, based on those answers, you could get help from the AI to take a closer look at some of those parts, you would have a better assessment. And I think that would have actually benefited the entire health center”.

P03—Psychologist

However, participants raised concerns about potential inaccuracies in AI's interpretation of language and non-verbal cues, particularly in digital triage, which might lead to overdiagnosis or misdiagnosis. Young adults undergoing significant life changes were considered particularly vulnerable to such inaccuracies. Furthermore, participants worried that relying on AI to define mental health problems might undermine human interactions, stigmatize young adults, or erode clinicians' autonomy when AI-generated conclusions lacked contextual human reflection. A balanced AI-human collaboration was deemed essential to ensure person-centered care.

“This is being able to interpret symptoms. And I think as a nurse, it's not just that easy … You have to be able to read between the lines. And there I see a risk with systems that only consider the text. Because sometimes patients write … If you only think about mental illness and suicide risk, sometimes they don't write that “I want to kill myself”. But it can be worded a little discreetly or you can understand it so that “the way this patient writes, I can't rule that out”. I think that there may be a risk there that one can miss symptoms that are serious”.

P05—Nurse

#### 3.1.2 Patient values and preferences

The participants emphasized the need for AI to support the assessment of patient satisfaction, alignment with patient preferences, and intrinsic motivation to follow through with care. AI was seen as a tool to accommodate individual preferences, such as whether to access care physically or digitally and to clarify patients' personal values for tailoring interventions to their needs.

“Motivation for change, if you could pick it up. It can be depression, it can be anxiety. In other words, how motivated is the patient to actually carry out X and Y intervention or an assignment at home and what can be a sensible outcome based on these”.

P03—Psychologist

#### 3.1.3 Healthcare professional knowledge and recommendations

Healthcare professionals identified a strong need for AI-driven decision support to enhance treatment recommendations, particularly for multi-morbid cases. AI was seen as a potentially valuable tool for suggesting stepwise interventions, providing individualized advice based on lifestyle factors and risks, and providing recommendations during triage or pre-care stages to enable early recommendations and interventions.

“I think there's also an opportunity linked to when working with sleep therapy where patients have to be registered, I think that an AI could certainly help to pick out the appropriate intervention based on that … we work with a sleep diary anyway, and try to draw conclusions from that but what … Is it sleep restriction I should work with or is it stimulus control or is it relaxation exercises or something like that?”

P04—Psychologist

Concerns revolved around the potential loss of clinical autonomy, trust issues when communicating AI-based recommendations to patients, and discrepancies between AI suggestions and real-world contexts. Participants also questioned accountability if AI-driven recommendations result in patient harm. Maintaining transparency and human agency in decision-making was seen as critical.

“When it comes to mental illness, it's … I think that it's always the patient who is responsible for his or her own life. I can't force anyone to make changes, and neither can any AI. But then on the other hand, the AI may not have a psychologist's license … if the patient goes home and kills himself. So yes, that's a great question. Had that blame been put on the healthcare services then?”

P14—Psychologist

#### 3.1.4 Arranging follow-up

Participants envisioned AI to potentially play a valuable role in assessing the follow-up processes at three stages: triage, the conclusion of consultation sessions, and between-session monitoring. AI could help prioritize and schedule initial healthcare visits during triage by identifying the primary issue and matching patients to the most relevant care. At the end of consultation sessions, AI could assist in scheduling follow-ups by considering the needs, preferences, and availability of both patients and healthcare professionals, fostering a collaborative approach. Between sessions, AI could assess patient status and determine the necessity and type of follow-up care.

“Since we don't want weekly contacts but are a little more flexible so … “Now we've decided on on this, how long do we need?” and discuss it a little together with the patient. And there I think that an AI could provide great support just because “okay, together with the AI we assess that it would be good to see each other again soon”. And I think that it's always good to be able to lean back on as a therapist, that it's not just my gut feeling we're using here, but this is based on something. So I think it could be helpful”.

P04—Psychologist

Concerns included the risk of patients being misdirected to inappropriate care, undermining trust in the healthcare services. Additionally, over-reliance on AI for follow-ups might lead patients to become passive, expecting the system to initiate contact. Healthcare professionals also noted that not all young adults might accept the use of AI in their care or that they may not have the technical digital tools that allow them to access AI support. Despite these concerns, the majority of participants emphasized the importance of integrating AI to enhance scheduling processes while maintaining individual autonomy.

“it creates a frustration for all patients who end up in the wrong place. Many people get very frustrated … “now I've answered a lot of questions here and then I come to you and you say that you can't help me, that I need to contact my health center”. It also creates frustration and a lack of trust in the health care system”

P05—Nurse

### 3.2 Ideal elements

Healthcare professionals' reflections were on three of the four ideal elements of SDM: (1) unbiased information, (2) presenting evidence, and (3) defined roles.

#### 3.2.1 Unbiased information

Participants highlighted the potential for AI to support more objective and less biased assessments by providing visualized insights and alleviating the subjectivity inherent in mental health evaluations.

“I think that right now they make their own subjective assessment there, that “this patient probably only needs four treatment sessions” or “this one needs eight” or something like that. But it's a random choice based on my previous experience. So it would probably have been very helpful to be able to predict that”.

P14—Psychologist

#### 3.2.2 Presenting evidence

Participants viewed AI as a potentially valuable tool for presenting evidence-based information, particularly in prevention and early intervention efforts. They emphasized that AI could be beneficial if it serves as a starting point for discussions about preventive strategies, guiding decisions, and setting future care directions by providing scientifically grounded insights.

“if you start collecting data already from the children's care center up to their school age, there's probably a lot to find out. But, also getting concrete suggestions about which interventions we should actually go in for. Sometimes it's a bit trendy, that “now we're going for this”, young people in school, or something like that, but then you don't really have the evidence for … You may still have evidence that there's a problem … It needs to be spot on and right timing for it to have any effect, preventive interventions … a system with certain data, that you call certain people for everything from health checks, to early calls if you notice something”

P06—Nurse

#### 3.2.3 Defined roles

A critical concern emerged regarding the ambiguity of responsibilities when using AI-derived predictions. Specifically, participants questioned who should communicate predictive evidence about future mental health risks to young adults, particularly when these interactions might occur outside traditional healthcare settings. This lack of clarity risks complicating decision-making processes at various points of care.

“but it perhaps also requires more competence of those of us who work. I don't know, even if one gets this, sure we can sit here and guess and think that now it's like this, or it will probably happen … But, I guess we get there when we sit with our patients and we have this in our lap and then we say “This is what I see for you now”. Should I be the one to give that message then, or?”

P07—Nurse

### 3.3 General elements

Healthcare professionals' perspectives encompassed eight of the ten general elements (1) flexibility and individualized approach, (2) information exchange, (3) involving at least two people, (4) mutual respect, (5) partnership, (6) patient education, (7) patient participation. (8) process/stages.

#### 3.3.1 Flexibility and individualized approach

Participants emphasized the potential of AI to foster tailored and flexible care in mental healthcare. They highlighted the desire for AI utility that can help in adapting cognitive behavioral therapy (CBT) and psychoeducation programs to match young adults' individual knowledge levels and needs. Participants thought that AI could make these programs more efficient by allowing healthcare professionals to adjust or skip unnecessary steps. Additionally, they proposed AI-supported triage systems to replace rigid forms with more dynamic and qualitative assessments that can improve capturing individual patient needs and better in supporting decision-making.

“We talk a lot about step-by-step care because our resources are limited, we can’t give the entire treatment battery to all people, it would be a huge waste of resources and not everyone needs it. But just finding … It would be very good if you could find the ones who are good or benefit from a minor intervention? We are doing what is called psychoeducation, that is, teaching people “what is depression? Why does it occur? What are the symptoms like? What can you do to feel better and prevent a relapse and things like that”. And yes, In this step-by-step care, … If you could get computer help there, to see “what steps should we have? Which steps should we offer to which patients?”

P02—Psychologist

However, participants expressed concerns about losing the “human factor” when AI plays an intermediary role to improve flexibility. They worried that potential excessive reliance on AI might weaken the therapeutic alliance, reduce direct patient-therapist interaction, and jeopardize patient safety. There was also apprehension about AI reinforcing stereotypes by assuming homogeneity within populations, thus potentially stigmatizing young adults based on background characteristics.

“we may not always fit into these square boxes. “They are like that, and they are like that…” We are all unique as individuals, and it's important that you do not lose sight of this either, but that you actually … You remember it. That AI is a part, but then also this human factor that we also talk about, it also needs to be included. There are also circumstances around, and I think you have to be aware of them and remember. Otherwise, I think it can go too far, if you just completely rely on it”.

P08—Social worker

#### 3.3.2 Information exchange

The participants emphasized the need for AI to facilitate the exchange of both medical and personal information that is relevant to the case. They underscored the potential value of AI in helping healthcare professionals understand patients' expectations and preferences, enabling more meaningful communication and tailored care strategies. They highlighted the importance of conveying the most relevant information and also including the patients' worries and lifestyle events. However, concerns emerged around data privacy and disconnected or poorly designed AI systems that might increase stress or complicate workflows.

“It's something we ask for in our telephone interviews, expectations that the patient has. “How do you think this will work? Can you set aside time for conversations once a week? Can you do your home assignment in between?” How they imagine the way in which the help will be given and what they are prepared to sacrifice themselves, so to speak, to feel better. That is quite important. If they think of conversation or medicine, they think of … yes. Can they imagine a group, can they imagine … So, some questions like that … How the treatment should be done. You need a picture not only of the symptoms, but of the person as well, in some way. What does the person think, what does the person expect, what is the person prepared to do to feel better?”

P02—Psychologist

#### 3.3.3 Involving at least two people and mutual respect

AI was seen as a potential enabler of non-physical access to care, ensuring that two-way interaction remains possible even when patients cannot or do not want to attend in person. Participants emphasized the importance of AI adopting a non-stigmatizing approach toward young adults while respecting healthcare professionals' expertise. However, there were concerns that replacing human interaction with AI could lead to diminished care quality, and reduced patient trust.

“just this that something may be lost when there is no physical person sitting in front of you. However, the human factor can sometimes cause problems. And if the alliance doesn't work, it can work the other way. But that there is still perhaps a risk that something interpersonal disappears, and how does that effect the results of the care?”

P11—welfare officer

#### 3.3.4 Partnership and patient participation

The participants valued AI's ability to support partnerships and encourage patient participation by automating time-consuming tasks, thus allowing more time for human interaction. They emphasized that AI could also enhance collaboration by providing accessible information to build mutual understanding between patients and healthcare professionals. However, societal and institutional barriers, such as inadequate integration of young adults' lifestyles or mental states into AI systems, could hinder these efforts. Additionally, excluding healthcare professionals or patients from the AI development process was seen as a potential threat to fostering collaborative partnerships. In relation to patient participation, there were concerns that the lack of human interaction could result in young adults feeling unseen or unacknowledged, which could negatively impact their willingness to open up and hinder the effectiveness of the care efforts.

“This thing about confidence and trust, I think is also one of these issues that is most often created in human relationships, that it's one human to another. That you may not gain trust and confidence in a computer in the same way. And I think that in order to feel good, it's usually human contacts that most people need, and also to be confirmed and to be seen”.

P05—Nurse

#### 3.3.5 Patient education

AI was perceived as a valuable tool for identifying and delivering relevant psychoeducational content to young adults. Participants saw potential in AI to guide navigation through CBT and psychoeducation materials according to the young adults' individual needs, streamlining care processes and improving patient education outcomes.

“Much of our treatment is based on psychoeducation, and there can be I can imagine that AI might take the form of more individual psychoeducation. There is a lot of psychoeducation available digitally. But what if an AI, that … which in some way psychoeducational answers that if I as a young person communicate with AI that “when I came home today, I had a lot of anxiety that I didn't recognize”. AI can respond to that in some clever way … The AI has some kind of idea of “then you need” … Then you need to talk to someone, do you need to do a relaxation exercise, do you need to take a walk, something helping, individually. If the AI somehow knows me, knows who I am”.

P15—Welfare officer

#### 3.3.6 Process and stages

Participants saw AI as a potential solution to reduce the gap between primary and specialized care by improving healthcare navigation. They illustrated that AI would be particularly valuable if it could predict and clarify the next steps in the care process, reducing inefficiencies and ensuring smoother transitions for patients.

“My experience is that there's often a gap between BUP [Child and Adolescent Psychiatry] and psychiatry, that … well, when those patients are referred to seeking help at the health center first, and then they usually bounce back and forth between the health center and psychiatry, and that is problematic in itself, I think, so it really would … A lot could be done there”.

P01—Psychologist

## 4 Discussion

This study revealed that healthcare professionals recognized both the benefits and limitations of integrating AI-based decision support systems into mental healthcare within an SDM framework. Needs and concerns related to 15 out of the 23 SDM elements were brought up in the interviews, encompassing the essential, ideal, and general SDM categories. Overall, the findings underscore the potential dual role of AI as both facilitator and disruptor of SDM. While the healthcare professionals recognized that AI can enhance clinical decision-making and streamline care processes, they emphasized that its successful integration depends on addressing key concerns related to accuracy, accountability, trust, and the preservation of person-centred care. From the healthcare professionals perspectives, AI could add value throughout the continuum of mental healthcare for young adults e.g., supporting triage, treatment, administrative processes, psychoeducation and self-care. Importantly, participants emphasized the need to meaningfully incorporate patients' perspectives at multiple stages of AI use in SDM. This includes designing open-ended triage processes that better capture patients' narratives, offering tailored recommendations, and involving patients in decisions. Taken together, these findings highlight the importance of developing AI systems through collaborative processes that involve healthcare professionals, patients, and system designers, to ensure alignment with SDM principles. Such insights remain underexplored in the current literature (35).

### 4.1 The role of AI to enhance mental healthcare and shared decision-making

One of the key advantages of AI is its ability to assist in the assessment of common mental health problems and provide recommendations, either by evaluating the current state or by predicting future risks based on existing risk factors (52), which were aspects also brought up by participants in this study. Significant efforts are underway to develop AI models capable of predicting conditions such as depression, anxiety, and other mental health disorders before they manifest (53, 54). To ensure that such systems effectively support SDM, Abbasgholizadeh et al. (35) emphasize the importance of incorporating explainability, interpretability, reproducibility, and a human-centered design approach in the development of AI tools.

Participants in this study underscored the critical need for incorporating patients' perspectives at various stages of AI use in SDM. This includes employing open-ended triage processes to better capture patients' voices, providing patient-specific recommendations, and considering patient preferences in follow-up scheduling decisions. However, integrating such inclusion raises potential challenges and risks, such as the influence of a patient's health condition on their perceptions, privacy concerns (55) that may hinder patients from openly sharing their perspectives, and skepticism from both healthcare professionals and patients regarding the trustworthiness of AI systems to accurately convey needs and preferences (25, 55, 56).

The findings suggest that AI could contribute to more objective and less biased assessments by visualizing data and reducing the subjectivity inherent in mental health evaluations. Such functionality has the potential to strengthen SDM by giving both clinicians and patients a clearer and more consistent evidence base from which to explore options and make informed choices. However, participants in this study also voiced concerns that AI might inadvertently reinforce stereotypes by assuming homogeneity within populations, which could stigmatize young adults based on background characteristics. This dual potential is reflected in previous research, which highlights AI's capacity both to mitigate clinician bias and to promote quality of care (23, 57), while also warning of its risk to reproduce or exacerbate inequities if trained on biased or non-representative data (58, 59). From a health equity perspective, embedding transparency and ongoing critical reflection into human–AI decision-making is essential, not only to safeguard against unintended harms, but also to use AI's potential to make SDM in mental healthcare more inclusive and equitable.

The study also identified a gap in the participants' perceptions of the essential SDM elements, particularly in terms of addressing the needs and concerns related to AI's role in presenting options, discussing patient ability and self-efficacy, and ensuring clarity and understanding. Practices related to these SDM elements are vital for enhancing the therapeutic alliance and fostering self-management (60, 61). AI systems have the potential to personalize options and assess a patient's ability and understanding through feedback loops (62, 63). However, if not carefully implemented, AI-driven options and feedback mechanisms could negatively impact SDM by introducing opaque “black-box” recommendations or undermining healthcare professionals' and patients' autonomy. This could shift the dynamic away from human-centered approaches and toward paternalistic AI systems, ultimately reducing trust and collaboration (24, 27).

### 4.2 Integrating AI into mental health care for shared decision making

The integration of AI into clinical processes for SDM demands careful planning to ensure the inclusiveness of all stakeholders. As the traditional dual relationship between patients and healthcare professionals is evolving into a triad of partnership with AI-based decision support systems (64). Challenges arise in maintaining person-centered care, current research highlights a gap in understanding how AI can be effectively implemented to support SDM while preserving its core principles (35). Joseph-Williams et al. (65) suggest that the SDM steps can be distributed across multiple mediators, such as therapists, nurses, and AI systems, to enhance workflow efficiency. However, an over-reliance on multiple mediators risks reducing patient engagement by diluting the personal connection with individual caregivers.

AI has the potential to enhance SDM by improving flexibility, fostering partnerships, and facilitating information exchange. The participants in this study highlighted the need for individualized approaches, particularly in psychoeducation and triage processes. AI-powered conversational agents, while promising in these areas (66, 67), face challenges related to safety and reliability, as generative systems can sometimes provide inappropriate responses. Ensuring high predictability and safety remains critical for deploying these technologies in healthcare.

Information exchange dynamics in SDM can shift the paradigm between paternalism and consumerism, depending on the flow and direction of information (68). The participants expressed a need for both medical and personal information to flow seamlessly between patients and providers via AI support. However, barriers such as low trust in AI-guided information and anxiety over potential errors must be addressed. Addressing these concerns requires strategies to preserve patient autonomy, foster human-AI collaboration, and train healthcare professionals on integrating AI into workflows (69–71).

There was a gap regarding the participants’ perspectives on AI to support key SDM elements, such as deliberation, negotiation, and reaching a middle ground. AI may disrupt these interactions, shifting SDM dynamics toward either extreme (64). This dynamic interplay between patients, healthcare professionals, and AI can vary depending on whether all three or only two parties interact at a time, potentially complicating SDM processes (45). Future research should explore healthcare professionals' perceptions of AI's role in facilitating balanced SDM conversations to inform implementation strategies and optimize outcomes.

### 4.3 Strengths and limitations

Involving a snowball sampling procedure of healthcare professionals with various professional backgrounds with experience in mental healthcare for young adults, alongside the researchers’ extensive experience in the methodology, facilitated an in-depth analysis and strengthened the credibility of the findings (46). As this study employed qualitative content analysis, the focus was on capturing the breadth and depth of experiences rather than comparing responses by professional role. The number of participants within each group precluded subgroup analysis, as conducting such analysis could have posed a risk to participant anonymity. Thus, the study did not distinguish the specific needs and concerns of individual professional group. Instead, we analyzed data from the collective perspective of healthcare professionals, aiming to identify shared and diverse views across roles. To enhance transparency, participant professions are noted in selected quotations.

A limitation of this study could be that the interview questions did not explicitly address SDM and that we did not specifically inquire about participants' formal training or knowledge of SDM practices. This omission means we cannot definitively assess the extent of participants' practical and theoretical understanding of SDM. However, existing research in the mental health field indicates that many healthcare professionals have limited formal training or knowledge of SDM practices (72, 79). This suggests that our participant group, consisting of healthcare professionals in mental health care, may share similar gaps in knowledge. This may have influenced how they conceptualized the role of AI in supporting decision-making, as their interpretations of SDM could vary. Nonetheless, the aim of this study was to interpret participants' reflections through the lens of an SDM framework, in order to explore how their experiences and views align with core elements of SDM.

The aim of qualitative content analysis is to generate in-depth understanding and nuanced insights into participants’ perspectives and previous research supports that our sample size is appropriate for qualitative analysis (73, 74). The richness of the data and the diversity of views expressed by the participating healthcare professionals contributed meaningfully to the exploration of AI integration in SDM. Furthermore, recurring perspectives and patterns emerged across interviews, indicating that the sample captured a broad range of relevant views and provided a solid foundation for the analysis.

During the data analysis, we followed the guidance of Graneheim et al. (46), emphasizing reflexivity and researcher dialogue as key strategies for ensuring analytical rigor in qualitative research, rather than relying on inter-coder reliability measures. One potential limitation of not employing formal inter-coder reliability is the possibility of subjective bias, as individual interpretations of the data may differ. However, this concern was mitigated through a collaborative and iterative process involving continuous dialogue, critical reflection, and consensus-building among the authors. Our interdisciplinary research team, bringing together expertise in mental health, SDM, AI, and qualitative methodology, further enriched the analytical process and enhanced the credibility and trustworthiness of the findings.

This study used Makoul and Clayman's (48) integrative model of shared decision-making (SDM) as a framework to explore AI's potential to support elements of SDM within mental healthcare for young adults. However, the findings primarily captured participants' perspectives on isolated elements of the SDM process, rather than the broader, integrated process as a whole. This represents a limitation, as effective SDM depends on the seamless interplay between multiple interconnected components. Consequently, while the study provides insights into how AI could support specific aspects of SDM, it does not offer a comprehensive understanding of how AI might facilitate or enhance the full SDM process. Nevertheless, the framework served as a valuable tool for identifying key element where AI could enhance SDM, underscoring its potential to improve both the quality and implementation of SDM through AI-based decision support systems.

### 4.4 Implications for practice and future research

This study provides a foundational step toward understanding AI's role in practical SDM models. By capturing healthcare professionals' initial perspectives on the integration of AI into SDM practice, the study provides valuable insights into how AI can support, enhance, and potentially transform decision-making processes in mental health care. Furthermore, it lays the groundwork for future research efforts aimed at exploring the real-world implementation of AI-enabled SDM, with a particular focus on identifying facilitators and addressing barriers to its adoption in clinical practice (35).

Reflecting on the challenges identified in this study, it is important to consider their potential implications for the youth population and for the practical design and implementation of AI systems targeting this group. Young adults are particularly vulnerable to the rapid changes and complexities posed by technology (75, 76). This study suggests that future AI services should avoid removing the human element from the interaction, as this may risk losing an essential therapeutic dimension such as the experience of being heard, listened to, and communicated with in humane way. Furthermore, to prevent the exacerbation of existing inequalities among young adults, attention needs to be given for ensuring the accessibility to the educational and decisional benefits that AI can provide.

Previous research has also highlighted the challenges and complications of using AI to support decision-making in healthcare context (24–26). Considering this, it is crucial in the design of AI systems for young adults to prioritize their safety, privacy, and well-being, guided by ethical guidelines that prevent exploitation and harm (77, 78). Responding both to ethical imperatives and to the limitations noted in prior literature, this study implies that AI systems need to be designed to support the positive integration between young adult's lifestyle and healthcare access. Such integration can enhance shared learning experiences between young adults and healthcare professionals, supporting SDM, promoting personalized mental healthcare, and strengthening the inclusion of the social context. Moreover, actively involving young adults in the design process can help ensure that their needs and perspectives are adequately addressed, leading to more effective and inclusive AI-based decision support systems.

The findings underscore the importance of co-designing AI solutions with end-users, addressing concerns about excluding healthcare professionals and patients from development processes. This aligns with previous research emphasizing the need for stakeholder involvement in AI design (25, 69). Future studies should involve young adults and healthcare professionals in the co-designing of AI-based decision support systems to align with end-user needs and practical SDM realities. This study identifies specific AI utilities that can inform system development, ensuring solutions are user-centered and contextually relevant.

Additionally, the study highlights specific SDM elements where AI can be impactful, such as triage, risk factor analysis for prevention, and patient psychoeducation. However, less emphasis was placed on elements like presenting options and fostering mutual agreement, warranting further investigation into healthcare professionals' perspectives to address these gaps.

Eight out of the 23 SDM elements were not emphasized by the participants. Among the essential elements of SDM, presenting options, discussing options pros/cons, discussing patient ability, checking clarity and patient understanding, and finalizing a decision were not brought up by the participants. Similarity, needs and concerns related to mutual agreement, deliberation/negotiation, and reaching a middle ground in decisions were not emphasized by participants within the ideal and general SDM elements. Several factors may explain these absences. First, the study design encouraged participants to reflect broadly on their needs and concerns regarding AI in decision support, rather than prompting them to consider each SDM element in detail. Second, participants' roles outside specialized care, together with potentially limited practical familiarity with AI and SDM, may have shaped the scope of their reflections. This finding suggests important avenues for future research, particularly studies that more explicitly examine these elements, to better understand how AI systems can be designed to support the less-emphasized SDM elements and thereby strengthen SDM in mental healthcare.

## 5 Conclusions

This study contributes to a deeper understanding of healthcare professionals' perspectives on integrating AI into SDM within mental healthcare for young adults. The healthcare professionals identified AI's potential to enhance early problem identification, triage, patient participation, information exchange, psychoeducation, and self-care, supporting a more personalized, participatory, and integrated approach to care. The healthcare professionals also emphasized the opportunity for AI to support the inclusion of patients' perspectives throughout various stages of the SDM process. This included designing open-ended triage systems that better capture patients' narratives, accommodating individual preferences, facilitating tailored information provision, and involving patients in care decisions. At the same time, healthcare professionals raised concerns about the potential overreliance on AI, which could undermine trust, reduce human interaction, and negatively affect patient safety. Additional risks were identified in relation to data privacy, equity in access, and increased demands on healthcare systems. These insights underscore the importance of designing AI tools in close collaboration with both healthcare professionals and young adults to ensure alignment with the theoretical foundations of SDM in mental healthcare.

As the integration of AI into SDM in mental healthcare for young adults remains a developing and underexplored field, further research is needed in several key areas. Priorities include gaining a deeper understanding of young adults' perspectives and examining how AI tools are implemented in real-world settings, as well as their effects on patient participation, quality of care, and clinical outcomes. Expanding this knowledge is crucial to ensure that future AI solutions are effectively aligned with the needs and values of both young adults and healthcare professionals.

## Data Availability

The original contributions presented in the study are included in the article/Supplementary Material, further inquiries can be directed to the corresponding author.
