# Supplementary material for: Healthcare Professionals’ perspectives on AI-driven decision support in young adult mental health: an analysis through the lens of a shared decision-making framework
Source: Front Digit Health. 2025 Sep 25;7:1588759. doi: 10.3389/fdgth.2025.1588759 (PMC12507755; doi:10.3389/fdgth.2025.1588759)
Supplement: Supplementary file 1 [file Table1.pdf]

Supplementary Table 1. The SDM framework as the theoretical foundation for the deductive analysis in the study.

SDM elements	Definition and clarification
1. Essential elements	
Define/explain problem	A step in the decision-making process where the healthcare professional and the patient collaboratively identify the mental health condition or issue to be addressed and for the healthcare professional to articulate/communicate it to the patient or young adult, ensuring mutual understanding and clarity.<br><br>(Makoul & Clayman, 2006; Sandman & Munthe, 2010)
Present options	The step of providing or presenting treatment options, or care direction choices between the healthcare professional and the patient, ensuring clarity and accessibility of available options.<br><br>(Edwards & Elwyn, 2009)
Discuss pros/cons (benefits/risks/costs)	The step of presentation of the benefits, risks, and costs of different options/directions of care or potential consequences upon acting on treatment options, specific care decision, or care direction choices.<br><br>(Edwards & Elwyn, 2009)
Patient values/preferences	The step of identification the patient's perspective including their beliefs, values, interests and preferences in relation to care decisions or treatment options, to ensure that the chosen option/decision/direction aligns with what matters most to the patient.<br><br>(Edwards & Elwyn, 2009; Elwyn et al., 2012)
Discuss patient ability/self-efficacy	The step of assessment and communication of the patient's ability by the healthcare professional or perceived ability by the patient, asserting the patient's capacity over executing the potential chosen option or when discussing the choices in hand.<br><br>(Bandura, 1990)
[α] Healthcare professional knowledge/recommendations	The communication of medical or professional processed information in the form of knowledge or recommendation that can be presented to the healthcare professional or the patient to support a specific option, care direction, or decision. (E.g.: Recommendation for treatment option, or ..etc.)

	(Edwards & Elwyn, 2009; Frické, 2009)
Check/clarify understanding	The step of confirming that the patient understands the options, or the choices clearly.<br>(Makoul & Clayman, 2006)
Make or explicitly defer decision	The step of deciding/finalizing a decision or option to be acted on.<br>(Makoul & Clayman, 2006)
Arrange follow-up	The step of coordination, and scheduling of a healthcare visit or next follow-up to continue with care process.<br>(Makoul & Clayman, 2006)
2. Ideal elements	
Unbiased information	The approach of providing information that is accurate, objective, and free from personal or systemic biases.<br>(Edwards & Elwyn, 2009; Ozdemir & Finkelstein, 2018)
Define roles (desire for involvement)	The step of communicating and understanding of healthcare professional's and patient's roles and desires to be included or responsible for a specific step in the decision-making process.<br>(Charles et al., 1997)
Present evidence	The approach of providing evidence-based backup for the options, discussions, or information that will be used to make a decision. I.e.: Supporting the information with evidence.<br>(Towle et al., 1999a)
Mutual agreement	The efforts of reaching or facilitate reaching consent for a chosen option, decision, or a care direction by both the patient and the healthcare professional.<br>(Aggarwal & Dupont, 2001)
3. General qualities	

Deliberation/negotiation	An approach of reflective process in which the patient, or both the patient and healthcare professional, consider medical and personal factors, including available options and what matters most to the patient (e.g., values, preferences, and abilities). This approach involves back and forth discussions to achieve a thorough understanding of both medical perspectives and the patient's perspective, ultimately enabling the patient to articulate their preferred direction of care.<br><br>(Charles, Gafni, et al., 1999; Edwards & Elwyn, 2009; Elwyn et al., 2012)
Flexibility/individualized approach	The approach of adapting the decision-making process to the patient's unique context, needs, and changing circumstances. An approach to decision-making that supports a flexible care process, adjusting to the healthcare professional's expertise and the patient's values and preferences. The approach emphasizes tailoring options and the care process to align closely with the patient's needs, abilities, values, and personal interests.<br><br>(Charles, Gafni, et al., 1999; Légaré & Witteman, 2013)
Information exchange	The process that allows two-way of sharing of information between patient and healthcare professionals, to serve the SDM purposes.<br><br>(Edwards & Elwyn, 2009; Makoul & Clayman, 2006)
Involves at least two people	An approach/process of involving two individuals working together to determine a specific decision or direction of care.<br><br>(Charles et al., 1997)
Middle ground	An approach/process of decision-making in healthcare that balances the roles of the healthcare professional (HCP) and the patient, avoiding extremes of paternalism (where the HCP is the sole decision-maker) and consumerism (where the patient independently makes decisions). This approach emphasizes shared responsibility, where both parties engage in the decision-making process, integrating the HCP's expertise and the patient's values and preferences to arrive at a mutually agreed-upon decision.<br><br>(Joseph-Williams, Edwards, et al., 2014; Wensing et al., 2002)
Mutual respect	An approach that unconditionally values both the healthcare professional and the patient as individuals, respecting their autonomy and acknowledging their perspectives, regardless of the two individual's thoughts or feelings about each other's opinions regarding the mutual decision in hand.

	i.e.: Ensuring that patient and healthcare professionals respect each other's expertise and preferences.<br><br>(Beach et al., 2007)
Partnership	An approach of positioning the healthcare professionals and patients as collaborators with a shared goal to decide upon a care related decision with the purpose of improving the patient's health.<br><br>(Charles, Whelan, et al., 1999; Towle et al., 1999b)
Patient education	An approach that aims to provide accessible, understandable information to enhance the patient's knowledge about their condition, healthcare journey and options.<br><br>(Edwards & Elwyn, 2009; Nicholson Thomas et al., 2017; Schuele et al., 2014)
Patient participation	An approach that empowers, motivates, and facilitates patient participation and inclusion in the decision-making process, ensuring their perspectives, preferences, and values are actively considered and integrated into care decisions.<br><br>(Fraenkel & McGraw, 2007)
Process/Stages	An approach of splitting/iterating SDM into different steps or phases. The overall processes can be managed by single or multiple healthcare professionals responsible to guide the patient through the SDM steps.<br><br>(Elwyn et al., 2012; Joseph-Williams, Elwyn, et al., 2014a)

[α]: In Makoul and Clayman (2006), the element originally labeled "Doctor Knowledge/Recommendations" has been adjusted to "Healthcare Professional Knowledge/Recommendations" to reflect that SDM can involve a multi-consultation model, including "non-doctor" healthcare professionals such as nurses, social workers, and others, in addition to doctors.

References

Aggarwal, V. K., & Dupont, C. (2001). Negotiation and Bargaining: Organizational Aspects. In *International Encyclopedia of the Social & Behavioral Sciences* (pp. 10473–10477). Elsevier. https://doi.org/10.1016/B0-08-043076-7/04301-1

Bandura, A. (1990). Perceived self-efficacy in the exercise of control over AIDS infection. *Evaluation and Program Planning*, *13*(1), 9–17. https://doi.org/10.1016/0149-7189(90)90004-G

Beach, M. C., Duggan, P. S., Cassel, C. K., & Geller, G. (2007). What Does 'Respect' Mean? Exploring the Moral Obligation of Health Professionals to Respect Patients. *Journal of General Internal Medicine*, *22*(5), 692–695. https://doi.org/10.1007/s11606-006-0054-7

Charles, C., Gafni, A., & Whelan, T. (1997). Shared decision-making in the medical encounter: What does it mean? (or it takes at least two to tango). *Social Science & Medicine*, *44*(5), 681–692. https://doi.org/10.1016/S0277-9536(96)00221-3

Charles, C., Gafni, A., & Whelan, T. (1999). Decision-making in the physician–patient encounter: Revisiting the shared treatment decision-making model. *Social Science & Medicine*, *49*(5), 651–661. https://doi.org/10.1016/S0277-9536(99)00145-8

Charles, C., Whelan, T., & Gafni, A. (1999). What do we mean by partnership in making decisions about treatment? *BMJ*, *319*(7212), 780–782. https://doi.org/10.1136/bmj.319.7212.780

Edwards, A., & Elwyn, G. (2009). Shared decision-making in health care: Achieving evidence-based patient choice. In A. E. Elwyn Glyn (Ed.), *Shared Decision-*

Making in Health Care (pp. 3–10). Oxford University PressOxford. https://doi.org/10.1093/oso/9780199546275.003.0001

Elwyn, G., Frosch, D., Thomson, R., Joseph-Williams, N., Lloyd, A., Kinnersley, P., Cording, E., Tomson, D., Dodd, C., Rollnick, S., Edwards, A., & Barry, M. (2012). Shared Decision Making: A Model for Clinical Practice. *Journal of General Internal Medicine*, *27*(10), 1361–1367. https://doi.org/10.1007/s11606-012-2077-6

Fraenkel, L., & McGraw, S. (2007). What are the Essential Elements to Enable Patient Participation in Medical Decision Making? *Journal of General Internal Medicine*, *22*(5), 614–619. https://doi.org/10.1007/s11606-007-0149-9

Frické, M. (2009). The knowledge pyramid: A critique of the DIKW hierarchy. *Journal of Information Science*, *35*(2), 131–142. https://doi.org/10.1177/0165551508094050

Joseph-Williams, N., Edwards, A., & Elwyn, G. (2014). Power imbalance prevents shared decision making. *BMJ*, *348*(may14 7), g3178–g3178. https://doi.org/10.1136/bmj.g3178

Joseph-Williams, N., Elwyn, G., & Edwards, A. (2014a). Knowledge is not power for patients: A systematic review and thematic synthesis of patient-reported barriers and facilitators to shared decision making. *Patient Education and Counseling*, *94*(3), 291–309. https://doi.org/10.1016/j.pec.2013.10.031

Joseph-Williams, N., Elwyn, G., & Edwards, A. (2014b). Knowledge is not power for patients: A systematic review and thematic synthesis of patient-reported barriers and facilitators to shared decision making. *Patient Education and Counseling*, *94*(3), 291–309. https://doi.org/10.1016/j.pec.2013.10.031

Légaré, F., & Witteman, H. O. (2013). Shared Decision Making: Examining Key Elements And Barriers To Adoption Into Routine Clinical Practice. *Health Affairs*, *32*(2), 276–284. https://doi.org/10.1377/hlthaff.2012.1078

Makoul, G., & Clayman, M. L. (2006). An integrative model of shared decision making in medical encounters. *Patient Education and Counseling*, *60*(3), 301–312. https://doi.org/10.1016/j.pec.2005.06.010

Nicholson Thomas, E., Edwards, L., & McArdle, P. (2017). Knowledge is Power. A quality improvement project to increase patient understanding of their hospital stay. *BMJ Quality Improvement Reports*, *6*(1), u207103.w3042. https://doi.org/10.1136/bmjquality.u207103.w3042

Ozdemir, S., & Finkelstein, E. A. (2018). Cognitive Bias: The Downside of Shared Decision Making. *JCO Clinical Cancer Informatics*, *2*, 1–10. https://doi.org/10.1200/CCI.18.00011

Sandman, L., & Munthe, C. (2010). Shared Decision Making, Paternalism and Patient Choice. *Health Care Analysis*, *18*(1), 60–84. https://doi.org/10.1007/s10728-008-0108-6

Schuele, M., Widmer, T., Premm, M., Criegee-Rieck, M., & Wickramasinghe, N. (2014). Improving knowledge provision for shared decision making in patient-physician relationships—A multiagent organizational approach. *2014 47th Hawaii International Conference on System Sciences*, 646–655. https://doi.org/10.1109/HICSS.2014.86

Towle, A., Godolphin, W., Greenhalgh, T., & Gambrill, J. (1999a). Framework for teaching and learning informed shared decision making Commentary:

Competencies for informed shared decision making Commentary: Proposals based on too many assumptions. *BMJ*, *319*(7212), 766–771. https://doi.org/10.1136/bmj.319.7212.766

Towle, A., Godolphin, W., Greenhalgh, T., & Gambrill, J. (1999b). Framework for teaching and learning informed shared decision making Commentary: Competencies for informed shared decision making Commentary: Proposals based on too many assumptions. *BMJ*, *319*(7212), 766–771. https://doi.org/10.1136/bmj.319.7212.766

Wensing, M., Elwyn, G., Edwards, A., Vingerhoets, E., & Grol, R. (2002). Deconstructing patient centred communication and uncovering shared decision making: An observational study. *BMC Medical Informatics and Decision Making*, *2*(1), 2. https://doi.org/10.1186/1472-6947-2-2
