# Supplementary material for: Healthcare Professionals’ perspectives on AI-driven decision support in young adult mental health: an analysis through the lens of a shared decision-making framework
Source: Front Digit Health. 2025 Sep 25;7:1588759. doi: 10.3389/fdgth.2025.1588759 (PMC12507755; doi:10.3389/fdgth.2025.1588759)
Supplement: Supplementary file 2 [file Table2.pdf]

Supplementary Table 2. Number of participants who identified needs or concerns related to each SDM element

**SDM element**	**Number of participants**
**1. Essential elements**	
Define/explain problem	15
Present options	0
Discuss pros/cons (benefits/risks/costs)	0
Patient values/preferences	4
Discuss patient ability/self-efficacy	0
Doctor knowledge/recommendations	13
Check/clarify understanding	0
Make or explicitly defer decision	0
Arrange follow-up	8
**2. Ideal elements**	
Unbiased information	3
Define roles (desire for involvement)	1
Present evidence	2
Mutual agreement	0
**3. General qualities**	
Deliberation/negotiation	0
Flexibility/individualized approach	10
Information exchange	8
Involves at least two people	4
Middle ground	0
Mutual respect	1
Partnership	9
Patient education	2
Patient participation	3
Process/stages	2
